# Dimethyl hydrazine-1,2-dicarboxyl­ate–triphenyl­phosphine oxide (1/1)

**DOI:** 10.1107/S160053681101991X

**Published:** 2011-06-04

**Authors:** Bogdan Doboszewski, James McGarrah, Alexander Y. Nazarenko, Fabio da Paixao Soares

**Affiliations:** a Departamento de Química, Universidade Federal Rural de Pernambuco, 52171-900 Recife, PE, Brazil; b Department of Chemistry, State University of New York, College at Geneseo, 1 College Circle, Geneseo, NY 14454, USA; c Chemistry Department, State University of New York, College at Buffalo, 1300 Elmwood Ave, Buffalo, NY 14222-1095, USA

## Abstract

In the crystal structure of the title compound, C_4_H_8_N_2_O_4_·C_18_H_15_OP, two triphenyl­phosphine oxide mol­ecules and two dimethyl hydrazine-1,2-dicarboxyl­ate mol­ecules are connected *via* N—H⋯O hydrogen bonds of moderate strength and are related *via* a twofold rotational axis. Weak C_ar_—H⋯ O contacts strengthen the crystal structure.

## Related literature

For the Mitsunobu reaction, see: Mitsunobu (1981 ▶); Hughes (1992 ▶), Swamy *et al.* (2009 ▶). For the structures of analogous compounds, see: Anderson *et al.* (1996 ▶); Héroux & Brisse (1997 ▶); Wang *et al.* (2007 ▶). For the synthesis of this and related compounds, see Doboszewski (1997 ▶, 2009 ▶).
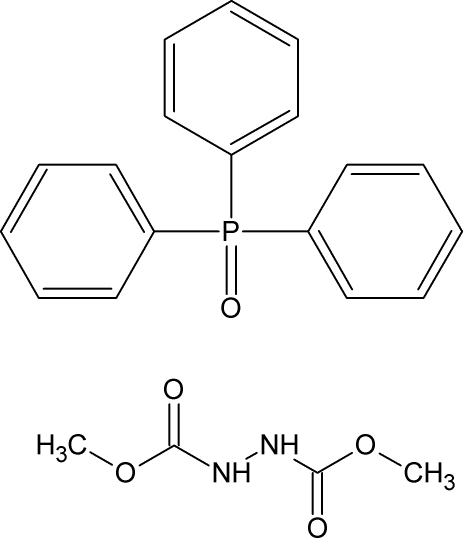


## Experimental

### 

#### Crystal data

C_4_H_8_N_2_O_4_·C_18_H_15_OP
*M* *_r_* = 426.39
Monoclinic, 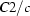

*a* = 26.494 (3) Å
*b* = 8.5545 (9) Å
*c* = 20.4426 (19) Å
β = 109.090 (3)°
*V* = 4378.3 (8) Å^3^
*Z* = 8
Mo *K*α radiation
μ = 0.16 mm^−1^
*T* = 200 K
0.8 × 0.7 × 0.4 mm

#### Data collection

Bruker SMART X2S diffractometer
Absorption correction: multi-scan (*SADABS* (Sheldrick, 2008*a* ▶) *T*_min_ = 0.84, *T*_max_ = 0.93
20468 measured reflections
3862 independent reflections
2956 reflections with *I* > 2σ(*I*)
*R*_int_ = 0.036

#### Refinement

*R*[*F* ^2^ > 2σ(*F* ^2^)] = 0.036
*wR*(*F* ^2^) = 0.095
*S* = 1.03
3862 reflections
298 parameters
H atoms treated by a mixture of independent and constrained refinement
Δρ_max_ = 0.26 e Å^−3^
Δρ_min_ = −0.30 e Å^−3^

### 

Data collection: *GIS* (Bruker, 2010 ▶); cell refinement: *APEX2* (Bruker, 2010 ▶) and *SAINT* (Bruker, 2009 ▶); data reduction: *SAINT* and *XPREP* (Bruker, 2009 ▶); program(s) used to solve structure: *SHELXS97* (Sheldrick, 2008*b* ▶); program(s) used to refine structure: *SHELXL97* (Sheldrick, 2008*b* ▶); molecular graphics: *ORTEP-3 for Windows* (Farrugia, 1997 ▶) and *Mercury* (Macrae *et al.*, 2008 ▶); software used to prepare material for publication: *PLATON* (Spek, 2009 ▶).

## Supplementary Material

Crystal structure: contains datablock(s) I, global. DOI: 10.1107/S160053681101991X/mw2011sup1.cif

Structure factors: contains datablock(s) I. DOI: 10.1107/S160053681101991X/mw2011Isup2.hkl

Supplementary material file. DOI: 10.1107/S160053681101991X/mw2011Isup3.cml

Additional supplementary materials:  crystallographic information; 3D view; checkCIF report

**Fig. 3.**
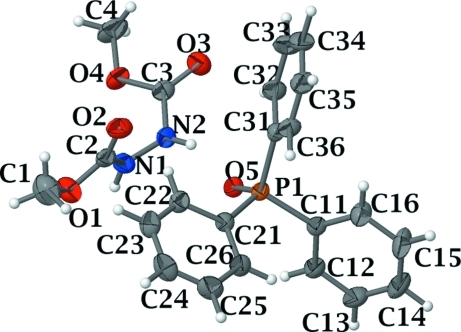
View of the title compound showing displacement ellipsoids at the 50% probability level.

Enhanced figure: interactive version of Fig. 3

## Figures and Tables

**Table 1:** Hydrogen-bond geometry (Å, °)

*D*—H⋯*A*	*D*—H	H⋯*A*	*D*⋯*A*	*D*—H⋯*A*
N1—H1⋯O5^i^	0.85 (2)	2.05 (2)	2.899 (2)	174.2 (19)
N2—H2⋯O5	0.83 (2)	2.05 (2)	2.833 (2)	155.9 (17)
C13—H13*A*⋯O2^ii^	0.92	2.53	3.310 (3)	143
C35—H35*A*⋯O3^iii^	0.98	2.54	3.270 (3)	131

Symmetry codes: (i) 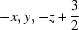; (ii) 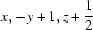; (iii) 
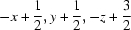
.

**Fig. 1.**
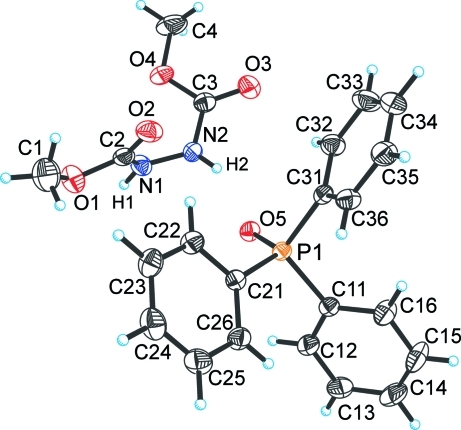
Perspective view of the dimethyl hydrazine-1,2-dicarboxylate - triphenylphosphine oxide adduct with displacement ellipsoids drawn at the 50% probability level.

**Fig. 2.**
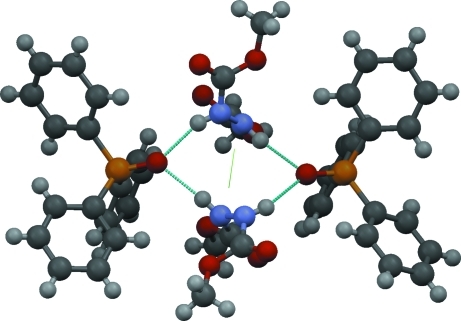
Two of the adducts connected via N—H···O hydrogen bonds.

## supplementary crystallographic information

### Comment

The two main by-products of the Mitsunobu reaction are bis-substituted hydrazines RO_2_CNHNHCO_2_R and phosphine oxides O=P*R*_3_. Both types of compounds can interfere with the isolation of the desired products as in our work using N3-benzoylthymine. In such cases it was necessary to skip isolation of the initially formed product and to perform debenzoylation in basic medium and eventually isolate the much more polar deprotected final compound. We recently alkylated N3-benzoylthymine and uracil with allyl and propargyl alcohol, Ph_3_P and *i*PrO_2_CNNCO_2_*i*Pr in dioxane or tetrahydrofuran for further transformations. After treatment of the crude reaction mixture with sodium methoxide in methanol, we isolated a crystalline compound identified by the present study as a 2:2 complex of the substituted hydrazine with triphenylphosphine oxide. Evidently, a transesterification occurred during the sodium methoxide treatment.

Even though the Mitsunobu reaction has been known for over 40 years, only in 1996 has an X-ray structure of a similar complex (Anderson *et al.*, 1996, Héroux & Brisse, 1997) been published and its stability was attributed to strong association *via* hydrogen bonding. The present communication shows a second compound of this kind.

In the adduct the bond lengths of the hydrazine molecule are the same within 0.01 Å as in the crystal structure of pure dimethyl hydrazine-1,2-dicarboxylate (Wang *et al.*, 2007) while the H—N—N—H torsion angle increases from 95 to 105° with adduct formation. This value is almost identical to that in the di(isopropyl) hydrazine-1,2-dicarboxylate adduct (Héroux & Brisse, 1997).

That there is little or no change in component geometry with adduct formation suggests that the hydrogen bonds formed are of only moderate strength (Table 1). This is also supported by the modest shift of the P=O stretching frequency from 1190 cm^-1^ in pure triphenylphosphine oxide to 1166 cm^-1^ in the adduct which is one property expected to be sensitive to the strength of the hydrogen bonding.

### Experimental

The compound is a by-product of the Mitsunobu reaction: allyl alcohol was employed for alkylation of thymine using Ph_3_P and *i*PrO_2_CNNCO_2_*i*Pr in dioxane. After treatment of the crude reaction mixture with sodium methoxide in methanol, a crystalline compound was isolated (m.p. 127–129° C). Parent peaks at 148 and 278 were clearly visible in a mass spectrum of the crystal dissolved in dichloromethane. Evidently, a transesterification occurred during a sodium methoxide treatment.

FTIR (Nicolet Nexus 470, Diamond ATR): 3229 and shoulder at 3180 (N—H), 3023 and 2952 (C—H), 1731 (C=O), 1535, 1434,1362,1263, 1225, 1165 and 1153, 116, 1068, 996,869,718, 694, 538 cm^-1^.

Raman (Raman Systems 2.0, 785 nm laser): 1737 (C=O), 1590, 1442, 1169, 997 (very strong), 871, 689, 618,531, 437, 300, 266 and 206 cm^-1^.

A comparatively large block was cut for the data collection due to the relatively low sensitivity of the CCD detector. Data collection was limited to θ=25° because of the geometry of the instrument.

### Refinement

All H atoms attached to C were positioned geometrically with *U*_iso_(H) = 1.2 or 1.5 *U*_eq_(C) and allowed to ride on the attached atom with C-H bond distances being free to refine (AFIX 44 and AFIX 138 constraints). The two hydrogen atoms of the hydrazine group which form hydrogen bonds with the phosphine oxide group were refined with isotropic displacement parameters. All C—H bonds are 0.90–0.98 Å, N—H bonds are 0.83 and 0.85 Å.

### Crystal data

**Table:**

C_4_H_8_N_2_O_4_·C_18_H_15_OP	*F*(000) = 1792
*M**_r_* = 426.39	*D*_x_ = 1.294 Mg m^−^^3^
Monoclinic, *C*2/*c*	Mo *K*α radiation, λ = 0.71073 Å
Hall symbol: -C 2yc	Cell parameters from 3730 reflections
*a* = 26.494 (3) Å	θ = 2.5–24.4°
*b* = 8.5545 (9) Å	µ = 0.16 mm^−^^1^
*c* = 20.4426 (19) Å	*T* = 200 K
β = 109.090 (3)°	Prism, colourless
*V* = 4378.3 (8) Å^3^	0.8 × 0.7 × 0.4 mm
*Z* = 8	

### Data collection

**Table:**

Bruker SMART X2S diffractometer	3862 independent reflections
Radiation source: XOS X-beam microfocus source	2956 reflections with *I* > 2σ(*I*)
doubly curved silicon crystal	*R*_int_ = 0.036
ω scans	θ_max_ = 25.1°, θ_min_ = 2.5°
Absorption correction: multi-scan (*SADABS* (Sheldrick, 2008*a*)	*h* = −31→31
*T*_min_ = 0.84, *T*_max_ = 0.93	*k* = −10→10
20468 measured reflections	*l* = −24→24

### Refinement

**Table:**

Refinement on *F*^2^	Primary atom site location: structure-invariant direct methods
Least-squares matrix: full	Secondary atom site location: difference Fourier map
*R*[*F*^2^ > 2σ(*F*^2^)] = 0.036	Hydrogen site location: inferred from neighbouring sites
*wR*(*F*^2^) = 0.095	H atoms treated by a mixture of independent and constrained refinement
*S* = 1.03	*w* = 1/[σ^2^(*F*_o_^2^) + (0.0533*P*)^2^] where *P* = (*F*_o_^2^ + 2*F*_c_^2^)/3
3862 reflections	(Δ/σ)_max_ = 0.001
298 parameters	Δρ_max_ = 0.26 e Å^−^^3^
0 restraints	Δρ_min_ = −0.30 e Å^−^^3^

### Special details

**Table:**

Geometry. All e.s.d.'s (except the e.s.d. in the dihedral angle between two l.s. planes) are estimated using the full covariance matrix. The cell e.s.d.'s are taken into account individually in the estimation of e.s.d.'s in distances, angles and torsion angles; correlations between e.s.d.'s in cell parameters are only used when they are defined by crystal symmetry. An approximate (isotropic) treatment of cell e.s.d.'s is used for estimating e.s.d.'s involving l.s. planes.
Refinement. Refinement of *F*^2^ against ALL reflections. The weighted *R*-factor *wR* and goodness of fit *S* are based on *F*^2^, conventional *R*-factors *R* are based on *F*, with *F* set to zero for negative *F*^2^. The threshold expression of *F*^2^ > σ(*F*^2^) is used only for calculating *R*-factors(gt) *etc*. and is not relevant to the choice of reflections for refinement. *R*-factors based on *F*^2^ are statistically about twice as large as those based on *F*, and *R*- factors based on ALL data will be even larger.

### Fractional atomic coordinates and isotropic or equivalent isotropic displacement parameters (Å^2^)

**Table:**

	*x*	*y*	*z*	*U*_iso_*/*U*_eq_	
P1	0.149303 (17)	0.57905 (5)	0.87467 (2)	0.02544 (14)	
O1	−0.00845 (5)	0.79421 (15)	0.65671 (7)	0.0488 (4)	
O2	0.07350 (5)	0.70178 (15)	0.66494 (6)	0.0447 (3)	
O3	0.08693 (5)	0.23349 (15)	0.65300 (7)	0.0476 (4)	
O4	0.01686 (5)	0.35822 (15)	0.57612 (6)	0.0451 (3)	
O5	0.10118 (4)	0.50237 (13)	0.82537 (5)	0.0294 (3)	
N1	0.00680 (6)	0.54291 (18)	0.67207 (8)	0.0378 (4)	
H1	−0.0244 (8)	0.535 (2)	0.6754 (10)	0.048 (6)*	
N2	0.03969 (6)	0.41275 (18)	0.68918 (8)	0.0372 (4)	
H2	0.0640 (7)	0.419 (2)	0.7272 (10)	0.036 (5)*	
C1	0.00936 (10)	0.9500 (2)	0.65050 (13)	0.0638 (7)	
H1C	−0.0205 (4)	1.0138 (9)	0.6276 (8)	0.096*	
H1B	0.0332 (6)	0.9482 (3)	0.6246 (8)	0.096*	
H1A	0.0271 (6)	0.9911 (9)	0.6953 (6)	0.096*	
C2	0.02861 (7)	0.6822 (2)	0.66519 (8)	0.0351 (4)	
C3	0.05118 (7)	0.3283 (2)	0.63982 (9)	0.0349 (4)	
C4	0.02347 (10)	0.2626 (3)	0.52124 (11)	0.0646 (7)	
H4C	0.0053 (6)	0.3099 (11)	0.4776 (6)	0.097*	
H4B	0.0089 (6)	0.1613 (14)	0.5229 (5)	0.097*	
H4A	0.0606 (5)	0.2535 (15)	0.5269 (5)	0.097*	
C11	0.16370 (7)	0.50014 (18)	0.96079 (8)	0.0281 (4)	
C12	0.12062 (8)	0.4739 (2)	0.98395 (10)	0.0405 (5)	
H12A	0.0857 (7)	0.4938 (5)	0.9542 (6)	0.049*	
C13	0.12841 (9)	0.4190 (2)	1.04989 (10)	0.0486 (5)	
H13A	0.0996 (6)	0.4028 (4)	1.0647 (3)	0.058*	
C14	0.17909 (9)	0.3886 (2)	1.09350 (11)	0.0517 (6)	
H14A	0.18455 (16)	0.3523 (9)	1.1397 (11)	0.062*	
C15	0.22194 (9)	0.4102 (3)	1.07079 (11)	0.0575 (6)	
H15A	0.2541 (8)	0.3883 (6)	1.0982 (7)	0.069*	
C16	0.21459 (8)	0.4665 (2)	1.00490 (9)	0.0425 (5)	
H16A	0.2438 (6)	0.4818 (4)	0.9902 (3)	0.051*	
C21	0.14139 (6)	0.78746 (18)	0.88138 (8)	0.0268 (4)	
C22	0.13023 (7)	0.8756 (2)	0.82090 (10)	0.0350 (4)	
H22A	0.12774 (8)	0.8242 (10)	0.7772 (8)	0.042*	
C23	0.12265 (8)	1.0355 (2)	0.82236 (11)	0.0422 (5)	
H23A	0.11515 (17)	1.0970 (12)	0.7799 (9)	0.051*	
C24	0.12570 (8)	1.1079 (2)	0.88404 (11)	0.0473 (5)	
H24A	0.12009 (15)	1.216 (2)	0.88489 (11)	0.057*	
C25	0.13689 (8)	1.0227 (2)	0.94401 (11)	0.0457 (5)	
H25A	0.13925 (9)	1.0737 (11)	0.9865 (9)	0.055*	
C26	0.14479 (7)	0.8620 (2)	0.94297 (10)	0.0352 (4)	
H26A	0.15250 (16)	0.8033 (11)	0.9845 (8)	0.042*	
C31	0.20790 (7)	0.55624 (18)	0.84924 (8)	0.0276 (4)	
C32	0.20893 (8)	0.4427 (2)	0.80066 (9)	0.0392 (5)	
H32A	0.1776 (6)	0.3766 (13)	0.7795 (4)	0.047*	
C33	0.25459 (8)	0.4243 (3)	0.78255 (11)	0.0515 (6)	
H33A	0.25523 (9)	0.3520 (18)	0.7511 (8)	0.062*	
C34	0.29896 (8)	0.5155 (3)	0.81221 (10)	0.0477 (5)	
H34A	0.3296 (7)	0.5006 (4)	0.8003 (3)	0.057*	
C35	0.29792 (8)	0.6288 (2)	0.85965 (10)	0.0423 (5)	
H35A	0.3295 (6)	0.6949 (14)	0.8805 (4)	0.051*	
C36	0.25267 (7)	0.6494 (2)	0.87778 (9)	0.0352 (4)	
H36A	0.25208 (7)	0.7255 (16)	0.9091 (6)	0.042*	

### Atomic displacement parameters (Å^2^)

**Table:**

	*U*^11^	*U*^22^	*U*^33^	*U*^12^	*U*^13^	*U*^23^
P1	0.0270 (2)	0.0250 (2)	0.0231 (3)	−0.00232 (18)	0.00640 (19)	−0.00216 (18)
O1	0.0400 (8)	0.0444 (8)	0.0621 (9)	0.0031 (6)	0.0167 (7)	0.0007 (7)
O2	0.0374 (8)	0.0594 (9)	0.0420 (8)	−0.0045 (6)	0.0192 (6)	−0.0049 (6)
O3	0.0497 (8)	0.0438 (8)	0.0514 (9)	0.0081 (7)	0.0195 (7)	0.0000 (6)
O4	0.0513 (8)	0.0565 (9)	0.0254 (7)	0.0014 (7)	0.0097 (6)	−0.0057 (6)
O5	0.0281 (6)	0.0295 (6)	0.0275 (6)	−0.0044 (5)	0.0049 (5)	−0.0050 (5)
N1	0.0282 (9)	0.0469 (10)	0.0385 (10)	0.0018 (7)	0.0113 (7)	−0.0041 (7)
N2	0.0327 (9)	0.0480 (10)	0.0259 (9)	0.0065 (7)	0.0026 (8)	−0.0050 (7)
C1	0.0649 (16)	0.0467 (14)	0.0768 (17)	−0.0003 (11)	0.0192 (13)	0.0018 (12)
C2	0.0311 (10)	0.0512 (12)	0.0218 (10)	0.0021 (9)	0.0070 (8)	−0.0043 (8)
C3	0.0353 (10)	0.0372 (11)	0.0337 (11)	−0.0056 (9)	0.0132 (9)	−0.0016 (8)
C4	0.0955 (19)	0.0660 (15)	0.0359 (13)	−0.0054 (13)	0.0262 (12)	−0.0144 (11)
C11	0.0327 (10)	0.0237 (9)	0.0267 (10)	−0.0028 (7)	0.0082 (8)	−0.0032 (7)
C12	0.0400 (11)	0.0510 (12)	0.0321 (11)	0.0040 (9)	0.0139 (9)	0.0054 (9)
C13	0.0553 (14)	0.0582 (14)	0.0403 (13)	0.0019 (10)	0.0267 (11)	0.0055 (10)
C14	0.0726 (17)	0.0516 (13)	0.0304 (12)	−0.0028 (11)	0.0164 (11)	0.0101 (9)
C15	0.0484 (13)	0.0737 (16)	0.0383 (13)	0.0008 (11)	−0.0024 (10)	0.0208 (11)
C16	0.0356 (11)	0.0528 (13)	0.0358 (12)	−0.0052 (9)	0.0072 (9)	0.0093 (9)
C21	0.0253 (9)	0.0258 (9)	0.0281 (10)	−0.0024 (7)	0.0069 (7)	−0.0027 (7)
C22	0.0389 (11)	0.0325 (10)	0.0332 (11)	−0.0022 (8)	0.0111 (8)	0.0003 (8)
C23	0.0470 (12)	0.0315 (10)	0.0449 (12)	−0.0027 (8)	0.0107 (9)	0.0071 (9)
C24	0.0483 (13)	0.0252 (10)	0.0635 (15)	−0.0010 (8)	0.0114 (11)	−0.0041 (10)
C25	0.0508 (13)	0.0366 (11)	0.0453 (12)	−0.0017 (9)	0.0096 (10)	−0.0170 (10)
C26	0.0385 (10)	0.0324 (10)	0.0310 (10)	−0.0018 (8)	0.0064 (8)	−0.0052 (8)
C31	0.0301 (9)	0.0279 (9)	0.0234 (9)	0.0005 (7)	0.0071 (7)	0.0016 (7)
C32	0.0411 (11)	0.0403 (11)	0.0368 (11)	−0.0021 (9)	0.0136 (9)	−0.0076 (9)
C33	0.0575 (14)	0.0564 (14)	0.0464 (13)	0.0051 (11)	0.0251 (11)	−0.0156 (10)
C34	0.0407 (12)	0.0643 (14)	0.0453 (13)	0.0059 (10)	0.0241 (10)	0.0021 (11)
C35	0.0348 (11)	0.0546 (12)	0.0391 (12)	−0.0064 (9)	0.0142 (9)	0.0012 (10)
C36	0.0375 (11)	0.0391 (10)	0.0306 (10)	−0.0042 (8)	0.0131 (8)	−0.0052 (8)

### Geometric parameters (Å, °)

**Table:**

P1—O5	1.4938 (11)	C14—C15	1.373 (3)
P1—C31	1.8017 (17)	C14—H14A	0.9582
P1—C21	1.8056 (16)	C15—C16	1.383 (3)
P1—C11	1.8058 (17)	C15—H15A	0.8729
O1—C2	1.342 (2)	C16—H16A	0.9271
O1—C1	1.433 (2)	C21—C26	1.388 (2)
O2—C2	1.203 (2)	C21—C22	1.395 (2)
O3—C3	1.209 (2)	C22—C23	1.384 (2)
O4—C3	1.347 (2)	C22—H22A	0.9782
O4—C4	1.445 (2)	C23—C24	1.383 (3)
N1—C2	1.352 (2)	C23—H23A	0.9780
N1—N2	1.386 (2)	C24—C25	1.373 (3)
N1—H1	0.85 (2)	C24—H24A	0.9379
N2—C3	1.354 (2)	C25—C26	1.391 (2)
N2—H2	0.832 (18)	C25—H25A	0.9546
C1—H1C	0.9492	C26—H26A	0.9485
C1—H1B	0.9492	C31—C36	1.389 (2)
C1—H1A	0.9492	C31—C32	1.396 (2)
C4—H4C	0.9539	C32—C33	1.385 (3)
C4—H4B	0.9539	C32—H32A	0.9810
C4—H4A	0.9539	C33—C34	1.375 (3)
C11—C16	1.385 (2)	C33—H33A	0.8962
C11—C12	1.389 (2)	C34—C35	1.378 (3)
C12—C13	1.378 (3)	C34—H34A	0.9307
C12—H12A	0.9422	C35—C36	1.377 (2)
C13—C14	1.372 (3)	C35—H35A	0.9830
C13—H13A	0.9187	C36—H36A	0.9161
			
O5—P1—C31	112.56 (7)	C15—C14—H14A	120.1
O5—P1—C21	112.97 (7)	C14—C15—C16	120.5 (2)
C31—P1—C21	105.14 (7)	C14—C15—H15A	119.7
O5—P1—C11	110.80 (7)	C16—C15—H15A	119.7
C31—P1—C11	108.28 (8)	C15—C16—C11	120.22 (19)
C21—P1—C11	106.73 (8)	C15—C16—H16A	119.9
C2—O1—C1	115.29 (16)	C11—C16—H16A	119.9
C3—O4—C4	115.36 (16)	C26—C21—C22	119.19 (16)
C2—N1—N2	118.65 (16)	C26—C21—P1	123.23 (13)
C2—N1—H1	122.3 (13)	C22—C21—P1	117.57 (13)
N2—N1—H1	117.6 (13)	C23—C22—C21	120.35 (18)
C3—N2—N1	121.10 (16)	C23—C22—H22A	119.8
C3—N2—H2	115.9 (12)	C21—C22—H22A	119.8
N1—N2—H2	114.3 (12)	C24—C23—C22	119.80 (19)
O1—C1—H1C	109.5	C24—C23—H23A	120.1
O1—C1—H1B	109.5	C22—C23—H23A	120.1
H1C—C1—H1B	109.5	C25—C24—C23	120.46 (19)
O1—C1—H1A	109.5	C25—C24—H24A	119.8
H1C—C1—H1A	109.5	C23—C24—H24A	119.8
H1B—C1—H1A	109.5	C24—C25—C26	120.04 (19)
O2—C2—O1	125.67 (18)	C24—C25—H25A	120.0
O2—C2—N1	125.44 (18)	C26—C25—H25A	120.0
O1—C2—N1	108.86 (15)	C21—C26—C25	120.16 (18)
O3—C3—O4	125.12 (17)	C21—C26—H26A	119.9
O3—C3—N2	122.87 (17)	C25—C26—H26A	119.9
O4—C3—N2	111.95 (16)	C36—C31—C32	119.02 (17)
O4—C4—H4C	109.5	C36—C31—P1	120.98 (13)
O4—C4—H4B	109.5	C32—C31—P1	120.00 (13)
H4C—C4—H4B	109.5	C33—C32—C31	119.46 (18)
O4—C4—H4A	109.5	C33—C32—H32A	120.3
H4C—C4—H4A	109.5	C31—C32—H32A	120.3
H4B—C4—H4A	109.5	C34—C33—C32	120.84 (19)
C16—C11—C12	118.54 (16)	C34—C33—H33A	119.6
C16—C11—P1	124.23 (14)	C32—C33—H33A	119.6
C12—C11—P1	117.23 (13)	C33—C34—C35	119.90 (19)
C13—C12—C11	120.77 (19)	C33—C34—H34A	120.0
C13—C12—H12A	119.6	C35—C34—H34A	120.0
C11—C12—H12A	119.6	C36—C35—C34	119.92 (19)
C14—C13—C12	120.1 (2)	C36—C35—H35A	120.0
C14—C13—H13A	119.9	C34—C35—H35A	120.0
C12—C13—H13A	119.9	C35—C36—C31	120.85 (18)
C13—C14—C15	119.8 (2)	C35—C36—H36A	119.6
C13—C14—H14A	120.1	C31—C36—H36A	119.6
			
C2—N1—N2—C3	−85.2 (2)	O5—P1—C21—C22	−58.38 (15)
C1—O1—C2—O2	−3.3 (3)	C31—P1—C21—C22	64.74 (15)
C1—O1—C2—N1	178.43 (16)	C11—P1—C21—C22	179.61 (13)
N2—N1—C2—O2	8.9 (3)	C26—C21—C22—C23	0.0 (3)
N2—N1—C2—O1	−172.85 (14)	P1—C21—C22—C23	178.68 (14)
C4—O4—C3—O3	2.5 (3)	C21—C22—C23—C24	−0.6 (3)
C4—O4—C3—N2	−174.72 (16)	C22—C23—C24—C25	0.8 (3)
N1—N2—C3—O3	165.73 (17)	C23—C24—C25—C26	−0.6 (3)
N1—N2—C3—O4	−17.0 (2)	C22—C21—C26—C25	0.3 (3)
O5—P1—C11—C16	138.93 (15)	P1—C21—C26—C25	−178.33 (14)
C31—P1—C11—C16	15.04 (17)	C24—C25—C26—C21	0.0 (3)
C21—P1—C11—C16	−97.70 (16)	O5—P1—C31—C36	163.98 (13)
O5—P1—C11—C12	−41.92 (15)	C21—P1—C31—C36	40.60 (16)
C31—P1—C11—C12	−165.81 (13)	C11—P1—C31—C36	−73.19 (15)
C21—P1—C11—C12	81.45 (14)	O5—P1—C31—C32	−16.60 (16)
C16—C11—C12—C13	1.5 (3)	C21—P1—C31—C32	−139.98 (14)
P1—C11—C12—C13	−177.73 (15)	C11—P1—C31—C32	106.23 (15)
C11—C12—C13—C14	−0.4 (3)	C36—C31—C32—C33	0.7 (3)
C12—C13—C14—C15	−1.2 (3)	P1—C31—C32—C33	−178.72 (14)
C13—C14—C15—C16	1.7 (3)	C31—C32—C33—C34	0.5 (3)
C14—C15—C16—C11	−0.7 (3)	C32—C33—C34—C35	−1.2 (3)
C12—C11—C16—C15	−0.9 (3)	C33—C34—C35—C36	0.7 (3)
P1—C11—C16—C15	178.22 (16)	C34—C35—C36—C31	0.5 (3)
O5—P1—C21—C26	120.24 (14)	C32—C31—C36—C35	−1.2 (3)
C31—P1—C21—C26	−116.65 (14)	P1—C31—C36—C35	178.21 (13)
C11—P1—C21—C26	−1.77 (16)		

### Hydrogen-bond geometry (Å, °)

**Table:**

*D*—H···*A*	*D*—H	H···*A*	*D*···*A*	*D*—H···*A*
N1—H1···O5^i^	0.85 (2)	2.05 (2)	2.899 (2)	174.2 (19)
N2—H2···O5	0.83 (2)	2.05 (2)	2.833 (2)	155.9 (17)
C13—H13A···O2^ii^	0.92	2.53	3.310 (3)	143
C35—H35A···O3^iii^	0.98	2.54	3.270 (3)	131

Symmetry codes: (i) −*x*, *y*, −*z*+3/2; (ii) *x*, −*y*+1, *z*+1/2; (iii) −*x*+1/2, *y*+1/2, −*z*+3/2.

## References

[bb1] Anderson, N. G., Lust, D. A., Colapret, K. A., Simpson, J. H., Malley, M. F. & Gougoutas, J. Z. (1996). *J. Org. Chem.* **61**, 7955–7958. 10.1021/jo9609539 11667761

[bb2] Bruker (2009). *SAINT* and *XPREP* Bruker AXS Inc., Madison, Wisconsin, USA.

[bb3] Bruker (2010). *GIS* and *APEX2* Bruker AXS Inc., Madison, Wisconsin, USA.

[bb4] Doboszewski, B. (1997). *Nucleosides Nucleotides*, **16**, 1049–1052.

[bb5] Doboszewski, B. (2009). *Nucleosides Nucleotides Nucleic Acids*, **28**, 875–901. 10.1080/15257770903306518 20183559

[bb6] Farrugia, L. J. (1997). *J. Appl. Cryst.* **30**, 565.

[bb7] Héroux, A. & Brisse, F. (1997). *Acta Cryst.* C**53**, 1318–1320.

[bb8] Hughes, D. L. (1992). *Org. React.* **42**, 335–656.

[bb9] Macrae, C. F., Bruno, I. J., Chisholm, J. A., Edgington, P. R., McCabe, P., Pidcock, E., Rodriguez-Monge, L., Taylor, R., van de Streek, J. & Wood, P. A. (2008). *J. Appl. Cryst.* **41**, 466–470.

[bb10] Mitsunobu, O. (1981). *Synthesis*, pp. 1–28.

[bb11] Sheldrick, G. M. (2008*a*). *SADABS* University of Göttingen, Germany.

[bb12] Sheldrick, G. M. (2008*b*). *Acta Cryst.* A**64**, 112–122. 10.1107/S0108767307043930 18156677

[bb13] Spek, A. L. (2009). *Acta Cryst.* D**65**, 148–155. 10.1107/S090744490804362X PMC2631630 19171970

[bb14] Swamy, K. C. K., Kumar, N. N. B., Balaraman, E. & Kumar, K. V. P. P. (2009). *Chem. Rev.* **109**, 2551–2651. 10.1021/cr800278z 19382806

[bb15] Wang, W., Taylor, C. M. & Fronczek, F. R. (2007). Private communication (refcode JIPFAQ). CCDC, Cambridge, England.

